# "You Travel Faster Alone, but Further Together": Learning From a Cross Country Research Collaboration From a British Council Newton Fund Grant

**DOI:** 10.15171/ijhpm.2018.73

**Published:** 2018-08-18

**Authors:** Priscilla Reddy, Rachana Desai, Sibusiso Sifunda, Kalipso Chalkidou, Charles Hongoro, William Macharia, Helen Roberts

**Affiliations:** ^1^Human Sciences Research Council, Population Health, Health Systems and Innovation, Cape Town, South Africa.; ^2^Faculty of Community and Health Science, University of the Western Cape, Cape Town, South Africa.; ^3^Human Sciences Research Council, HIV/AIDS, STI’s and TB, Pretoria, South Africa.; ^4^Centre for Global Development, London, UK.; ^5^School of Public Health, Imperial College London, London, UK.; ^6^Department of Surgery and Cancer, Centre for Global Development, London, UK.; ^7^Faculty of Health Sciences, Department of Paediatrics and Child Health, Aga Khan University, Nairobi, Kenya.; ^8^Faculty of Population Health Sciences, UCL Great Ormond Street Institute of Child Health, London, UK.

**Keywords:** Capacity Development, Workshop, Collaboration, Sustainability, Interdisciplinary

## Abstract

Providing universal health coverage (UHC) through better maternal, neonatal, child and adolescent health (MNCAH) can benefit both parties through North–South research collaborations. This paper describes lessons learned from bringing together early career researchers, tutors, consultants and mentors from the United Kingdom, Kenya, and South Africa to work in multi-disciplinary teams in a capacity-building workshop in Johannesburg, co-ordinated by senior researchers from the three partner countries. We recruited early career researchers and research users from a range of sectors and institutions in the participating countries and offered networking sessions, plenary lectures, group activities and discussions. To encourage bonding and accommodate cross-cultural and cross-disciplinary partners, we asked participants to respond to questions relating to research priorities and interventions in order to allocate them into multidisciplinary and cross-country teams. A follow up meeting took place in London six months later. Over the five day initial workshop, discussions informed the development of four draft research proposals. Intellectual collaboration, friendship and respect were engendered to sustain future collaborations, and we were able to identify factors which might assist capacity-building funders and organizers in future. This was a modestly funded brief intervention, with a follow-up made possible through the careful stewardship of resources and volunteerism. Having low and middle-income countries in the driving seat was a major benefit but not without logistic and financial challenges. Lessons learned and follow-up are described along with recommendations for future funding of partnerships schemes.

## Background

Health and social inequalities are increasing in low- and middle-income countries (LMICs)^1-3^ where maternal, neonatal, child and adolescent health (MNCAH) services are often fragmented, poorly co-ordinated and uneven in terms of quality and access. Inequalities of this kind result in high morbidity, premature mortality, over and under-nutrition, risk taking and poor mental health.^4-6^ Given the projected trajectories of neonatal, child, adolescent and maternal mortality rates,^7,8^ (Table action is required from both the north and the south to meet MNCAH Sustainable Development Goals (SDGs) by 2030.^3^ Tackling the global challenge of providing universal health coverage (UHC) requires an approach that brings together lay and professional producers and consumers of health and health services, researchers, programme implementers, and policy-makers.^9,10^

**Table:** Maternal, Neonatal, Infant and Child Mortality Rates in the Global North and South^7,8^

	**Life Expectancy at Birth, 2013 (y)**	**Neonatal Mortality Rate Per 1000 Live Births, 2016**	**Under-5 Mortality Rate Per 1000 Live Births, 2016**	**Adolescent (10-19 Years) Mortality Rate Per 100 000 Population, 2017**	**Maternal Mortality Ratio Per 100 000 Live Births, 2015**
South Africa	60	12.4	43.3	128.7	138
Kenya	61	22.6	49.2	206.8	510
UK	81	2.6	4.3	15.9	9
Africa region	58	27.2	76.5	242.6	545
European region	76	5.1	9.6	55.4	16

The Newton Fund is a part of the UK’s official development assistance (ODA) and supports research and innovation to address global issues affecting developing countries.^11^ It aims to build UK/partner country collaborations on the basis of shared challenges. The Researcher Links scheme is administered by the British Council and provides financial support to enable senior researchers to bring together cohorts of early career researchers to help build research partnerships and capacity.^12^

This paper describes experiences, challenges and lessons learned from bringing together early career researchers, tutors, consultants and mentors from the United kingdom, Kenya, and South Africa who had not previously worked together in a trilateral capacity building program. The aims were to contribute to capacity building and establish or strengthen research links with longer term sustainability through funding applications.

## Methods

The workshop built on an existing relationship between the South Africa and the United Kingdom and, through our networks, a third partner in Kenya was identified. Additional established researchers, in particular experienced African researchers and players in health policy and practice were identified to contribute, participate and act as mentors. These partners offered social and behavioural science expertise for tackling problems amongst the most excluded populations, health systems research, skills in trial design, health economics and evidence-informed policy-making. Front line clinical experience included primary health and paediatric care in rural areas and an extensive network of universities, hospitals and clinics. This helped us ensure that our work was firmly grounded in problems faced by service users and professionals in the ‘real world’ and not only research questions developed in elite institutions. The workshop leaders and mentors combined research with policy practice, clinical and funding expertise, and those selected for participation came from a range of relevant disciplines, united by shared interests in reducing inequalities in health.

Through our partner networks, we sought applications from early career researchers from a range of sectors and institutions in each country. Applicants were asked to describe their experiences in MNCAH, identify problems in their area of work and expectations of the programme. We received 111 applications, triaged using a British Council template, written information provided by participants, and selected 36 participants. Descriptions of all participants as well as leaders and mentors can be found here (https://drive.google.com/file/d/0B4tSZTKrvdAhX1RZckRhOHlFMmc/view?usp=sharing).

We sent applicants two pre-workshop tasks to enable us to match participants and mentors, and ensure cross-country dialogue. Firstly, they were encouraged to find a shared platform to communicate with one another and were introduced to online sharing modalities such as the Open Science framework, Dropbox, and Google Drive. Secondly, they were asked to respond briefly to four questions:

1. If there is one intervention I would like to test to improve MNCAH, it would be …
2. If there is one thing that has really improved things for MNCAH in my environment, it has been…
3. If there is one thing which we could stop doing it would be…
4. If you were to design an intervention, what would be your target population and what behaviour change strategy would you choose?

In addition to the internet and communication platforms for regular planning meetings prior to hosting the workshop, the use of communication platforms in the course of the workshop enabled global senior researchers to present and participate from a distance.

The workshop was held in South Africa and included plenary lectures on global issues in MNCAH, research methodologies, trilateral funding opportunities from the United Kingdom based ODA funding schemes, including a presentation by the British Council South Africa, group activities and general discussions. Interactive sessions covered qualitative, quantitative, policy and design thinking research approaches in the field of MNCAH, sharing case studies. Group sessions enabled team building and the development of concept papers in MNCAH. Participants joined one of four working teams under the guidance of a mentor. The teams worked together throughout the workshop to develop proposals. They were challenged to innovate and accommodate different research and dissemination approaches for optimal impact, and each presented their ideas to the whole workshop on the final day.

Following the workshop, mentors and organisers kept in touch through WhatsApp and email, and participants in the four groups continued to further develop their proposals for funding with support from leaders and mentors on request. An online portal for participants to share information and ideas was created on the Dropbox Platform.

As a result of careful financial stewardship by the South African hosts, there were savings on the grant which, with the agreement of the funders, was used for a follow-up meeting in London six months later. As before, the administrative effort from both the north and the south was on a voluntary basis, and included meeting space provided by the London academic hosts. While logistic, visa, timing and workload problems meant that attendance at this two day meeting was reduced, the use of Skype and written input in advance of, and during the meeting from those who could not be present enabled progress.

## Results and Discussion

The workshop was designed to provide an opportunity to explore common issues on MNCAH across the three countries and to partner and network in interdisciplinary teams. Unsurprisingly, the expectations of the workshop given by applicants at the point of application were very much in line with the ‘offer’ ie, a wish to collaborate, to learn, and to network. Once selected however, participants demonstrated the kind of constructive challenge which any good research group needs:

“*It is important to discuss at the workshop how reproductive choices are all too often made in the context of constraint, and interventions focused solely on behaviour change need to be embedded within broader structural changes in the design and delivery of maternity, maternal, and infant care services.”*

And challenging one of the pre-workshop questions, another participant suggested:

“*I would not choose a behaviour-change intervention. I would train and deploy more health workers, and support them to provide high-quality, respectful care. My target population would be the country’s most underserved areas. In addition to health worker shortages at the country-level, health workers tend to be unequally distributed within countries.*”

In the course of the workshop, we identified ways to maximise knowledge sharing through staff exchanges and interdisciplinary collaboration. Participants were given the tools to identify health system and research needs, and set up and expand monitoring processes for the health and social policy environment for achieving UHC in MNCAH in the United Kingdom, Kenya, and South Africa. Participants and their mentors worked together to design research protocols for understanding the implementation and impact of health technologies and social interventions for application in MNCAH services. The preliminary draft research proposals that emerged from our work were driven by participant enthusiasm and a willingness to forgo individual interests in pursuit of a common interest and were:

1. To assess the mental health impact of sexual/gender based violence among adolescents aged 13-19 years old in three urban areas in Kenya, South Africa, and the United Kingdom.
2. To understand Quality of Care from both patient and provider perspectives.
3. To evaluate the effectiveness and sustainability of hand-washing in schools in South Africa and Kenya.
4. To establish and evaluate community-based maternal and neonatal care resource centres for improved neonatal outcomes in low income populations.

Some of these proposals will fall by the wayside to be replaced by others in response to particular calls. Where there was no evident partner from (for instance) the United Kingdom, other contacts were suggested and contacted. Where appropriate, references were provided to the growing numbers of population-based randomized controlled trials (RCTs) in the United Kingdom, some of which might inform interventions in LMICs, with appropriate cautions on the importance of context and resources. As one participant put it in response to the pre-workshop task:

“*We need to stop implementing interventions that are not informed by any theory of change/monitoring and evaluation plan to determine effectiveness; implementing MNCAH interventions that are not designed and developed in collaboration with the intended beneficiaries.”*

Participants also identified low cost interventions which had originated in LMICS including Kangaroo Mother Care (KMC) and doulas which might be tested in the United Kingdom. In both cases, existing research evidence is important, but context matters.

The workshop and the responses to the pre-workshop tasks allowed us to identify both research strengths and development needs from all three participating countries and initiate collaborations. Promising aspects of the workshops include:

## A Willingness to Work Together Towards Multi-county Research Initiatives

Collaborations across countries have been identified, potential funders are being explored, and research bids are being drafted.

## Cross and Multi-disciplinary Approaches

The early career researchers and academics attending the workshop from the range of disciplines described above listened and learned from one another. Participants in clinical practice policy and funding ensured a ‘real world’ perspective.

## Theme and Content Issues

Common challenges included quality of care, and healthcare financing. Although the latter was most acutely described in Kenya and South Africa, it was by no means absent from discussions relating to the United Kingdom. There were lessons to be learned across countries, especially around financing where, for instance South Africa is still piloting aspects of its proposed National Health Insurance (NHI), whilst the UK’s National Health Service (NHS) established in 1948, and funded through taxation to be free at the point of need, is highly valued but continues to face challenges. Several Kenyans in their pre-workshop tasks identified positive changes in funding driven by political will.

## Mentoring and Capacity Development

Mentoring and capacity development emerged as critical areas to be enhanced and further developed through our network, creating a space for emerging researchers from the three countries to tap into the expertise of the established scientists who facilitated the workshop.

## Potential Future Collaborative Initiatives Across Countries

Core to this workshop were potential collaborations and future engagement of participants. Funding for capacity development and workshops continue to be explored by the project leaders and other participants. The South African and Kenyan teams are currently in discussions to conduct the first Youth Risk Behavior Survey (YRBS) amongst high school students in Kenya in 2018 – a study which has been conducted three times in South Africa between 2002 and 2011, and is widely cited. The YRBS aims to obtain nationally and provincially representative data on the prevalence of behaviors that place learners at risk.^13-15^

In addition to opportunities, there were constructive challenges:

- At times, differing scientific paradigms and goals were evident. Design thinking approaches, offered by consultants to the workshop, were felt by some to be helpful, whilst others found the approach unproductive.
- We had extensive discussions around the use of traditional Western based research methodologies in indigenous non-western cultural contexts.

Constraints included:

- A lack of dedicated financial support for administrative staff to plan the workshop, drawing instead on the voluntary resources of the host institutions in South Africa and London to manage, plan and organize the workshop – a time and resource-consuming activity.
- Eligibility criteria for applicants as stipulated by the funders was problematic. Participants were expected to be early career researchers who had been awarded a PhD not more than 10 years prior to the workshop, or near completion with an academic position (a permanent post, research contract, or fellowship) at a recognised research institution either in the United Kingdom, South Africa or Kenya. Whilst demand exceeded supply of places for UK applicants, this was less the case for Kenya and South Africa, where additional skills and expertise including governance, healthcare management, policy, and rural health were added to our inclusion criteria.
- Seed and pilot light funding can have an impact well beyond a relatively modest investment. That said, there are few mechanisms for monitoring longer term outcomes, which we expect to do as our group develops, changes and experiences success and failures.

The Newton Fund allowed us the flexibility to prioritise research needed in LMICs, while engaging high-income country (HIC) partners. This allowed the LMICs to take the major role of distributing and managing the fund, and leading research collaborations designed to maximise benefits and minimise harms.^16^ Recently, research capacity-building funders and partners have been criticised for not prioritising the research needs of developing countries, resulting in inequitable award of grants.^16,17^ Moreover, research institutions in middle income countries with pre-existing links with UK institutions have been found to be more likely to benefit from grant funding. Elite institutions in high income countries may be tempted or encouraged to seek out elite institutions in LMIC, potentially widening inequalities.

Going forward with our newly formed collaborations, lessons need to be learned from previous North-South collaborations. Van der Veken, Belaid, Delvaux, De Brouwere^18^ for example found a lack of time, resources, research skills, and donor influence for the choice of research topics were important factors. As highlighted in previous North-South collaborations, it is recommended that funds be reserved for the networks to initiate their own projects that emerge from the workshop with regular face-to face follow up meetings.^16-18^

In a positive step, in the United Kingdom a recent prosperity fund (https://www.gov.uk/government/collections/cross-government-prosperity-fund-programmes) review (the Fund has an allocation of around £80m for health alone), recommends that in future there is better coordination, targeting and strategic vision to avoid the fragmentation and potential dilution of aid’s primary objective, caused by its current “portfolio approach.”

In the light of the above, we consider that:

- There is a need for adequate funding to administer as well as run workshops of this kind.
- A mix of participants with practical and clinical experience alongside participants from academia is beneficial in steering the research proposals focused on MNCAH priorities.
- Funding for follow up in the medium to long term would be required to evaluate success and identify barriers to progress in workshops of this kind.
- Whilst LMIC researchers and clinicians appreciate a degree of direction from funders, strategic priorities need to be identified upfront with LMIC colleagues driving these on the basis of need. We are fortunate that this happened in our collaboration, but this is not universal.
- Building capacity where there is greatest need^17^ is an aspiration which could be further encouraged.

There are challenges in terms of culture, communication and follow up, some of which could be met by a training centre for research and practice excellence to tackle issues in MNCAH, sustain cross-country collaborations, identify resources and promote career development. The challenge of whether we did indeed travel further together than we might have done alone is one which can only be truly tested over time, but we can certainly identify green shoots of growth, and are continuing to do so.

## Acknowledgements

We would like to thank the NRF, Newton Fund and British Council for the financial support. We would also like to thank the participants and mentors voluntarily participating and attending the workshop.

## Ethical issues

Not applicable.

## Competing interests

This work was not sponsored by a funder with proprietary or financial interests in the outcome.

## Authors’ contributions

PR, KC, and WM are the grant holders for this workshop. They contributed to the conception and design of the workshop and manuscript. All authors participated in the workshop and contributed to the writing and editing of the manuscript.

## Authors’ affiliations

^1^Human Sciences Research Council, Population Health, Health Systems and Innovation, Cape Town, South Africa. ^2^Faculty of Community and Health Science, University of the Western Cape, Cape Town, South Africa. ^3^Human Sciences Research Council, HIV/AIDS, STI’s and TB, Pretoria, South Africa. ^4^Centre for Global Development, London, UK. ^5^School of Public Health, Imperial College London, London, UK. ^6^Department of Surgery and Cancer, Centre for Global Development, London, UK. ^7^Faculty of Health Sciences, Department of Paediatrics and Child Health, Aga Khan University, Nairobi, Kenya. ^8^Faculty of Population Health Sciences, UCL Great Ormond Street Institute of Child Health, London, UK.
